# Physiopathological Relevance of D-Serine in the Mammalian Cochlea

**DOI:** 10.3389/fncel.2021.733004

**Published:** 2021-12-17

**Authors:** Jing Wang, Nicolas Serratrice, Cindy J. Lee, Florence François, Jonathan V. Sweedler, Jean-Luc Puel, Jean-Pierre Mothet, Jérôme Ruel

**Affiliations:** ^1^Institute for Neurosciences of Montpellier (INM), University Montpellier, Institut National de la Santé et de la Recherche Médicale (INSERM), Montpellier, France; ^2^ENT Department, Hospital and University of Montpellier, Montpellier, France; ^3^Department of Chemistry, Beckman Institute, University of Illinois at Urbana-Champaign, Urbana, IL, United States; ^4^Laboratoire LuMin, Biophotonics and Synapse Physiopathology Team, Université Paris-Saclay, Centre National de la Recherche Scientifique (CNRS), ENS Paris Saclay, Centrale Supélec, Gif-sur-Yvette, France; ^5^Aix-Marseille Université, Centre National de la Recherche Scientifique (CNRS), Laboratoire de Neurosciences Cognitives, Marseille, France

**Keywords:** NMDA receptors, cochlea, acoustic trauma, neuroprotection, d-serine

## Abstract

NMDA receptors (NMDARs) populate the complex between inner hair cell (IHC) and spiral ganglion neurons (SGNs) in the developing and mature cochlea. However, in the mature cochlea, activation of NMDARs is thought to mainly occur under pathological conditions such as excitotoxicity. Ototoxic drugs such as aspirin enable cochlear arachidonic-acid-sensitive NMDAR responses, and induced chronic tinnitus was blocked by local application of NMDAR antagonists into the cochlear fluids. We largely ignore if other modulators are also engaged. In the brain, D-serine is the primary physiological co-agonist of synaptic NMDARs. Whether D-serine plays a role in the cochlea had remained unexplored. We now reveal the presence of D-serine and its metabolic enzymes prior to, and at hearing onset, in the sensory and non-neuronal cells of the cochlea of several vertebrate species. *In vivo* intracochlear perfusion of D-serine in guinea pigs reduces sound-evoked activity of auditory nerve fibers without affecting the receptor potentials, suggesting that D-serine acts specifically on the postsynaptic auditory neurons without altering the functional state of IHC or of the stria vascularis. Indeed, we demonstrate *in vitro* that agonist-induced activation of NMDARs produces robust calcium responses in rat SGN somata only in the presence of D-serine, but not of glycine. Surprisingly, genetic deletion in mice of serine racemase (SR), the enzyme that catalyzes D-serine, does not affect hearing function, but offers protection against noise-induced permanent hearing loss as measured 3 months after exposure. However, the mechanisms of activation of NMDA receptors in newborn rats may be different from those in adult guinea pigs. Taken together, these results demonstrate for the first time that the neuro-messenger D-serine has a pivotal role in the cochlea by promoting the activation of silent cochlear NMDAR in pathological situations. Thus, D-serine and its signaling pathway may represent a new druggable target for treating sensorineural hearing disorders (i.e., hearing loss, tinnitus).

## Introduction

N-methyl D-aspartate receptors (NMDARs) are major excitatory ligand-gated ion channels in the central nervous system (CNS), and are critical mediators for synaptic plasticity, learning, and memory. Native GluN1-GluN2 containing NMDAR are tetraheteromeric assemblies (Paoletti et al., 2013) whose activation require the binding of both L-glutamate and a co-agonist (Paoletti et al., 2013; Hansen et al., 2018). Although glycine was first proposed to serve as the coagonist of NMDARs (Johnson and Ascher, 1987; Forsythe et al., 1988; Kleckner and Dingledine, 1988) further evidence indicated that at many central excitatory synapses within different brain areas, the amino acid D-serine is the primary endogenous co-agonist of NMDARs (Mothet et al., 2000). Therefore, both D-serine and glycine can support NMDAR-dependent long-term changes in synaptic plasticity and cognitive processes (Li et al., 2013; Neame et al., 2019). Synthesis of D-serine is carried out by serine racemase (SR), an enzyme that converts L- into D-serine (Wolosker et al., 1999; Wolosker, 2018), while D-amino acid oxidase (DAAO), a peroxysomal enzyme, degrades D-serine (Pollegioni et al., 2018; Wolosker, 2018).

In striking contrast to the retina, where the role of D-serine has garnered extensive attention (Stevens et al., 2003; Dun et al., 2008; Kalbaugh et al., 2009; Sullivan et al., 2011), the functions of D-serine in other sensory organs remain enigmatic. The cochlea of the inner ear, however, is the primary structure for sound perception that conveys sound information to the brain and may provide an interesting model to examine the relative roles of D-serine and glycine. The cochlea houses the organ of Corti, which is made up of both supporting cells and mechanosensory hair cells whose primary function is the transduction of auditory signals to the central nervous system *via* chemical synapses on dendrites of spiral ganglion neurons. Indeed, the cochlear sensory inner hair cells (IHCs) use L-glutamate as a primary neurotransmitter to afferent type I auditory spiral ganglion neurons (SGNs) and use L-glutamate as a primary neurotransmitter for transforming acoustic information into electrical signals (Puel, 1995; Glowatzki et al., 2008; Fettiplace, 2020; Moser et al., 2020). SGNs express a variety of glutamate receptors types, including the NMDARs (Ruel et al., 2008; Zhang-Hooks et al., 2016), kainate (Fujikawa et al., 2014), and metabotropic receptors (Klotz et al., 2019), although fast glutamatergic neurotransmission is mainly conveyed by postsynaptic α-amino-3-hydroxy-5-methylisoxazole-4-propionic acid (AMPA) receptors (Ruel et al., 1999, 2000; Glowatzki and Fuchs, 2002; Sebe et al., 2017). Under physiological conditions, NMDARs are not functional at mature IHC-SGN synapses (Ruel et al., 1999; Glowatzki and Fuchs, 2002), despite the suggestions that they regulate surface expression of AMPARs (Chen et al., 2007; Sanchez et al., 2015), and that they participate in glutamatergic synaptic transmission in the developing cochlea by promoting spontaneous activity and survival of SGNs (Zhang-Hooks et al., 2016).

Conversely, NMDARs may be activated under pathological conditions such as drug- or sound-induced ototoxicity. Indeed, studies show that NMDARs promote neosynaptogenesis and cochlear ribbon synapse rearrangement at SGN dendritic terminals, enabling partial restoration of hearing after acute injury (D’Aldin et al., 1997). They are also involved in the genesis of salicylate-induced tinnitus (Guitton et al., 2003; Ruel et al., 2008). The evidence that NMDA receptors are activated after acoustic trauma comes from previous experiments conducted using the behavioral model of peripheral tinnitus in rats (Guitton et al., 2004). In addition, the therapeutic potential of cochlear NMDARs was tested in a randomized, double-blind clinical trial using an intratympanic injection of an NMDAR antagonist, on cochlear tinnitus caused by acute acoustic trauma (Van De Heyning et al., 2014).

However, the molecular mechanisms behind the pathological activation of NMDAR in the mature cochlea are not well understood. Activation of the NMDARs at the IHC-SGN ribbon synapse is thought to be primarily promoted by synaptic glutamate released from IHC (Zhang-Hooks et al., 2016), but whether a co-agonist such as D-serine or glycine may contribute or not remains unknown.

In the present study, we aimed to explore the functions of D-serine as an endogenous modulator of cochlear NMDARs, using several vertebrate species as models, and to characterize the potential mechanisms of action by which D-serine modulates cochlear neurotransmission. By combining analytical chemistry, molecular biology, immunocytochemistry, cochlea ultrastructural evaluation, *in vivo* electrophysiology, and calcium imaging, we demonstrate the presence of D-serine in the cochlea. We show that this unconventional amino acid plays a central role in the activation of silent cochlear NMDARs and that genetic deletion of D-serine, as occurring in serine racemase null-mutant mice, provides protection against noise-induced long term damage. Together, our results provide a new molecular mechanism for future therapeutic modulation of NMDARs, aiming at treating abnormal activation of NMDAR-induced hearing loss and tinnitus in humans.

## Materials and Methods

### Animals and Ethics Statements

Experiments were carried-out on three rodent species: mice, rats, and guinea pigs. Male serine racemase knockout (Ser^–/–^) mice and their heterozygous (Ser^+/–^) and wild-type (Ser^+/+^) littermates in the isogenic C57BL/6J genetic background were bred at the AnimEX facility (Université Paris-Saclay) and were 2 months old at the time of the experiments. Male adult Wistar rats and C57BL/6J mice were purchased from Janvier Laboratories (Le Genest Saint Isle, France) and used when 2 months old. One- to two-month-old tricolor adult guinea pigs were obtained from Charles River (L’Arbresle, France). Animals were housed on a 12 h light/dark cycle and were provided food and water *ad libitum*. The animals were bred in pathogen-free animal care facilities accredited by the French Ministry of Agriculture and Forestry (INM: C-34-172-36; December 19, 2014; AnimEX: C92-019-01). Experiments were conducted in accordance with European and French directives on animal experimentation and with local ethical committee approval (agreements C75-05-18 and 01476.02, license #6711).

### *In vivo* Perilymphatic Perfusion Technique

Adult pigmented guinea pigs, free of middle-ear infection, were anesthetized with urethane (1.4 g/kg i.p; Sanofi, France) and ventilated during the experiment. Supplemental doses (0.35 g/kg, i.p) were administered every 2 h, or more often if the animal withdrew its paw in response to deep pressure. A tracheotomy was performed, the electrocardiogram was monitored, and the core temperature was regulated at 38 ± 1°C by a regulated heating blanket. The pinna and external auditory meatus were resected to ensure good close-field acoustic stimulation.

The method used to monitor the effect of multiple perilymphatic perfusions on sound-evoked potentials has been extensively described previously (Puel, 1995). The cochlea was exposed ventrally, and two holes (0.2 mm in diameter) were gently drilled in the scala vestibuli and scala tympani of the basal turn of the cochlea. Intracochlear perfusions through the hole in the scala tympani flowed at 2.5 μl/min and exited the cochlea through the hole in the scala vestibuli. All perfusions lasted 10 min. The artificial perilymph solution had the following composition (in mM): 137 NaCl, 5 KCl, 2 CaCl_2_, 1 MgCl_2_, 1 NaHCO_3_, 11 glucose, and 10 HEPES (pH 7.4; 300 mOsm/kg H_2_O).

### Auditory Brainstem Response Recordings

Mice were anesthetized by an intraperitoneal injection of a mixture of Rompun 2% (3 mg/kg) and Zoletyl 50 (40 mg/kg). ABRs were recorded from three subcutaneous needle electrodes placed on the vertex (active), on the pinna of the tested ear, and in the neck muscles (ground) of the mice. The acoustical stimuli delivered at a rate of 10/s were generated by a NI PXI-4461 signal generator (National Instruments) and consisted of 10 ms tone-bursts with a 1 ms rise- and fall time. Sound was generated by a JBL 075 loudspeaker (James B. Lansing Sound) positioned at 10 cm from the tested ear in a calibrated free-field condition. Voltage amplification (20,000) was achieved *via* a Grass P511 differential amplifier, and responses were averaged 1,000 times (Dell Dimensions). Level-amplitude functions of the ABRs were obtained at each frequency tested (2, 4, 6.3, 8, 10, 12.5, 16, 20, 24, and 32 kHz) by varying the level of the tone bursts from 0 to 100 dB SPL, in 5 dB incremental steps. The ABR thresholds were defined as the minimum sound level necessary to elicit a well-defined and reproducible wave II.

### Compound Action Potential of the Auditory Nerve Recordings

CAP was elicited in adult pigmented guinea pigs using tone bursts generated by an arbitrary function generator (type 9100R; LeCroy Corporation) with a 1 ms rise/fall time and a 9 ms total duration. The signals were passed through a programmable attenuator and presented to the ear in the free field via a JBL 075 earphone. Functional assessment was realized at 8 kHz sound frequency, with increasing levels of 5 dB from 0 to 100 dB sound pressure level (SPL) (reference 2.10^–5^ Pa). The rate of presentation was 10 bursts per second. Cochlear responses were amplified (gain of 2,000) by a differential amplifier (Grass P511K; Astro-Med), and the signals filtered (bandpass, 100–3 kHz) and averaged (256 repeats) on a Pentium personal computer (Dell Computer Company). The sampling rate of the analog-to-digital converter was 50 kHz, with a dynamic range of 12 bits and 1,024 samples per record. CAPs were measured peak-to-peak between the first negative (N_1_) and the following positive value (P_1_). The thresholds were defined as the level needed to elicit a measurable response between 2 and 5 μV.

### Distortion Product Otoacoustic Emission Recordings

Distortion Product Otoacoustic Emissions (DPOAEs) were recorded in the external auditory canal using an ER-10C S/N 2528 probe (Etymotic research Inc., Elk Grove Village, IL, United States.). The two primary tones of frequency f1 and f2 were generated with a constant f2/f1 ratio of 1.2, and the distortion product 2f1-f2 processed by a Cubdis system HID 40133DP (Mimosa Acoustics Inc., Champaign, IL, United States). The probe was calibrated for the two stimulating tones before each recording. f1 and f2 were presented simultaneously, sweeping f2 from 20 to 2 kHz in quarter-octave steps. For each frequency, the distortion product 2f1-f2 and the neighboring noise amplitude levels were measured and expressed as a function of f2.

### *In vitro* Calcium Imaging in Spiral Ganglion Neurons

Postnatal 1–4-day old rat pups were decapitated and both inner ears removed from the base of the cranium for further dissociation. After the cochleae were extracted from the outer bony labyrinth, the outer ligament/stria vascularis and organ of Corti were dissected away from the central core of the cochlea that contained the spiral ganglion. The samples were then incubated with an enzymatic solution (collagenase type IV, 0.5 mg/ml and trypsin, 2.5 mg/ml) at 37°C for 1 h, shaken every 10 min. The enzymatic dissociation was stopped by a wash in a solution free of Ca^2+^ and Mg^2+^. Spiral ganglia were then dissociated mechanically using a Pasteur pipette whose tip had been flame-polished. The spiral ganglion neurons were centrifuged and then plated onto a poly-ornithine- and laminin-treated glass bottom dish (FluoroDish, World Precision Instrument, Inc., Sarasota, Florida, United States).

Two hours after plating, the spiral ganglion neurons were loaded with 3 μM fura-2 AM in a Locke buffer that had the following composition (in mM): 137NaCl, 3KCl, 2CaCl_2_, 1MgCl_2_, 10Glucose, 10Hepes, (pH = 7.35; osmolarity 300-303mOsm/kg H_2_O) at 37°C for 30 min. Images were acquired with a Cool SNAP CCD camera coupled to an inverted microscope (Axiovert 200; Zeiss). The neurons were illuminated at 340 nm and 380 nm with a monochromator (Lambda DG-4 Diaphot; Sutter Instruments, Burlingame, CA) controlled by the software Metafluor (Universal Imaging Corporation, West Chester, PA). Fura-2 fluorescence images were continuously acquired, giving 4–5 data points per second. The emitted fluorescence images were obtained through a dichroic mirror and an emission filter of 510 nm. After subtraction of the background obtained in a region of interest (ROI) where no neurons were present, the changes in intracellular calcium levels were monitored in neurons defined as ROI. The emission ratio (340/380 nm) was converted into Ca^2+^ concentrations using the equation developed by Grynkiewicz et al. (1985).

### Noise Trauma

SR^–/–^ mice (*n* = 6) and their corresponding wild-type (*n* = 6) counterparts aged at 2 months were placed under anesthesia [Rompun 2% (3 mg/kg) and Zoletil 50 (40 mg/kg)] into a small cage. The cage was suspended directly below the horn of the sound-delivery loudspeaker in a small, reverberant chamber. They were then exposed to noise in the 4–8 kHz band at a level of 100 dB SPL for 2 h. The noise was generated by a PCI 4461 generation card (National instruments) controlled by LabVIEW software. The sound level was calibrated before each exposure session using a 1/4-inch microphone (# 46BE, GRAS Sound and Vibration) controlled by a PCI 4461 card and LabVIEW software, ensuring that there was no more than a 1 dB difference between the center and the edge of the cage.

### *In vitro* Measurements of Amino Acid Content in Cochleae

1-month old SR^+/+^ mice (*n* = 8) were deeply anesthetized by i.p. injection of sodium pentobarbital (60 mg/kg body weight, Sanofi, Libourne, France), their cochleas were rapidly removed and placed individually in sterile eppendorf tubes in the presence of methanol. Extracted cochlea were then stored at −80°C until analysis. Cochlea were homogenized using a Precellys Evolution bead homogenizer. The supernatants were collected and dried using a Speedvac, and the pellets rinsed with 1,000 μL of purified water. The solution was combined with the original dried supernatant and evaporated to dryness in a Speedvac. All samples were reconstituted in purified water. Protein quantification was performed using the Micro BCA Protein Assay Kit (Thermo Fisher Scientific). The amino acids were pre-column derivatized with Nα-(2,4-Dinitro-5-fluorophenyl)-L-valinamide and analyzed with liquid chromatography coupled to tandem mass spectrometry (LC-MS/MS) to quantify D-serine, L-serine, glycine, and L-glutamate levels. This system used an EVOQ Elite Triple Quadrupole Mass Spectrometer coupled with an Elute UHPLC module (Bruker Daltonics) using a reversed-phase Kinetex phenyl-hexyl HPLC column [2.6 μm particle size, 100 Å pore size, 100 mm (length) × 2.1 mm ID (Phenomenex, Torrance, CA, United States)] with mobile phase A: 25 mM ammonium formate, mobile phase B: methanol, and flow rate 300 μL/min. Each analyte retention time and fragmentation parameter were established from the corresponding standard. The resulting chromatograms were analyzed by Data Reviewer 8 (Bruker Daltonics).

### Western-Blot Analysis

Brain and cochleae were homogenized in sample buffer [0.1 M NaCl, 20 Mm Tris, 5 Mm EDTA, 1 Mm PMSF, 1% SDS, with Complete protease inhibitor cocktail (Roche)]. Protein extracts (15 μg/lane) were separated on 10% (vol/vol) SDS-polyacrylamide gel and electro-blotted onto nitrocellulose membranes (0.45 μm, Thermo Fisher Scientific). Blots were incubated overnight at 4°C with goat polyclonal antibody against a peptide mapping the C-terminus of mouse SR (1/2,000, Santa Cruz Biotechnology, United States), or a rabbit polyclonal antibody against porcine kidney DAAO (1/1,000, Nordic Immunological Laboratories, The Netherlands). β-actin (1/10,000, Sigma-Aldrich) served as a loading control. The secondary antibodies used were horseradish peroxidase-conjugated donkey anti-goat IgG antibodies (1/3,000, Jackson ImmunoResearch Laboratories, United States) or donkey anti-rabbit IgG antibodies (1/3,000, Jackson ImmunoResearch Laboratories, United States). Immunodetection was accomplished by enhanced chemiluminescence (ECL) using the WesternBright Sirius femtogram-detection reagent (advansta). Molecular sizes were estimated by separating pre-stained molecular weight markers (10–260 kDa) in parallel (Fisher Scientific). Digital images of the chemiluminescent blots were captured using the chemidoc system (Biorad) with the Labimage acquisition software. To ascertain the expression of SR and DAAO in the cochlea, as a positive control, we used brain tissues known to express high levels of SR and DAAO (Verrall et al., 2007). Demonstration of the specificity of the polyclonal anti-SR antibody was achieved by using protein brain extracts from SR^–/–^ and WT (SR^+/+^) control mice (Supplementary Figure 2). Western-blot analysis required 12 cochleae per species. All experiments were performed in triplicate. All results were normalized by β-actin expression.

### Immunostains

**FIGURE 1:**
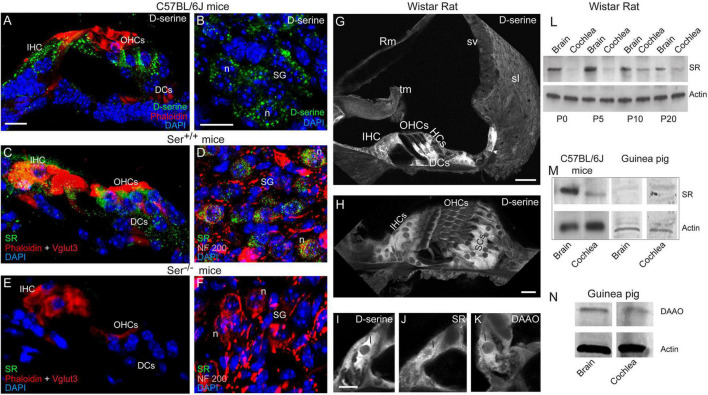
Expression and localization of D-serine, SR and DAAO in mouse, rat and guinea-pig cochleae. **(A–F)** Confocal images showing the cryostat transversal sections of the basal region of the cochleae from C57BL/6J **(A,B)**, Ser ^+/+^ **(C,D)** and Ser ^–/–^ **(E,F)** mice. The sections were immunolabeled for D-serine (green in **A,B**) or serine racemase (SR, green in **C–F**), Vglut3 (red in **C,E**) and NF 200 (red in **D,F**) to identify the inner hair cells and spiral ganglion neurons, respectively, rhodamine-phalloidin to identify actin (red in **A,C,E**) and counterstained with DAPI to stain the nuclei (blue). **(G–K)** Confocal images showing the vibratome transversal sections of the basal region of adult rat organ of Corti immunolabeled for D-serine **(G–I)**, SR **(J)** and DAAO **(K)**. Scale bars: **(A,H,I)** = 10 μm, **(BG)** = 20 μm. **(L–N)** Representative western blot analysis using antibodies against SR, DAAO, and β-actin in whole cochlear extracts from P0 to P20 rat cochlea and brain **(L)**, adult mouse, and guinea-pig cochlea and brain **(M,N)**. IHCs, inner hair cells; OHCs, outer hair cells; DCs, Deiters cells; Hcs, Hensen’ cells; Rm, Reissner’s membrane; tm, tectorial membrane; sv, stria vascularis; sl, spiral ligament; I, inner hair cell; SCs, supporting cells; sg, spiral ganglion; n, neuron.

Adult rat cochleae were prepared according to a standard protocol for fixation and embedding (Dememes et al., 2006). Cochlear slices of 60–80 μm were obtained using a vibratome and collected in ice-cold 0.1 M phosphate buffered saline (PBS). Adult mouse cochlear cryostat sections were prepared (Ladrech et al., 2004), and immunofluorescence procedures carried out as previously described (Dememes et al., 2006). After pre-incubation in the blocking buffer containing 10% normal donkey serum and 0.3% Triton X-100, sections were incubated at 4°C overnight with the primary antibodies including a rabbit polyclonal antibody raised against D-serine cross-linked to BSA with glutaraldehyde (GemacBio, Cenon, France) and diluted in PBS at 1/1,000 for D-serine, goat polyclonal antibody raised against the C-terminal part of the mouse SR (Santa Cruz Biotechnology, United States) diluted at 1/200, or a rabbit polyclonal antibody against porcine kidney DAAO (Nordic Immunological Laboratories, The Netherlands) diluted at 1/500. Anti-Vglut3 (Synapic Systems, #135204, RRID:AB_2619825) diluted at 1/500 and anti-neurofilament (NF 200, Sigma-Aldrich #N0142 RRID:AB-477257) diluted at 1/600 were used to identify the inner hair cells and spiral ganglion neurons, respectively. Rhodamine-conjugated phalloidin (1:1,000, Sigma) was used to label actin. After three PBS rinses, the cochleae were then incubated for 2 h at room temperature in PBS containing the secondary antibodies (Jackson ImmunoResearch Laboratories, PA, United States) diluted at 1/100 in PBS, as follows: Fluorescein isothiocyanate (FITC)-conjugated donkey anti-goat IgGs or FITC-conjugated donkey anti-rabbit IgGs and rhodamine-conjugated phalloidin (1/100 dilution; R-415, Molecular Probes) to double label for actin. DAPI (0.002% wt:vol, Sigma, Missouri, United States) was used to stain DNA. Fluorescence was acquired sequentially using a confocal microscope (LSM 5 Live Duo, Zeiss) with a 40x oil-objective, except for Figures 1E,H that were observed with a 10x-objective. Z stacks of optical sections were then projected onto a single plane using ImageJ and imported into Adobe Photoshop for adjustment of contrast and brightness. These optical sections were 0.25–0.5 μm thick, so that the reconstructed image roughly corresponded to a section thickness of 2.5–3 μm. In control specimens without primary antibodies, no FITC fluorescence signal was observed (Supplementary Figure 1). Immunocytochemistry analysis required 4 animals per species. All experiments were performed in triplicate.

### Transmission Electron Microscopy Analysis of the Organ of Corti

At the end of the *in vivo* electrophysiological session, cochleae were fixed by intracochlear perfusion of 3.5% glutaraldehyde in phosphate buffer (0.1 M, pH 7.4) through the perilymph compartment for 20 min, then removed and immersed in the same fixative overnight. The next day, fixed cochleae were rinsed in phosphate buffer and post-fixed in a 2% osmic acid solution for 1 h. After two rinses in phosphate buffer, the cochleae were dehydrated in a graded series of ethanol (30–100%), then in propylene oxide, and embedded in Epon resin at 60°C overnight. After trimming the cochleae to separate the different coils and to select samples of various positions along the organ of Corti (from base to apex), thin transverse sections (70–100 nm) were cut using a Leica-Reichert Ultracut E, counterstained with uranyl acetate and lead citrate, and observed using a transmission electron microscope (Hitachi 100) (*n* = 4 cochleae).

### Drugs

All chemicals and drugs were purchased from Sigma Chemical Company, except for AMPA, which was purchased from RBI and GYKI 53784 (LY303070) which was a generous gift of Margaret H. Niedenthal. Stock solutions were made and stored at -20°c before use, and final concentrations of the drug vehicle for GYKI in the bathing solution was kept at 0.4%.

### Statistics

Data were analyzed using MATLAB software. All values are expressed as means ± the standard error of mean (S.E.M) and were compared by the two-tailed Mann-Whitney Wilcoxon test or Student’s *t*-test. Significance was assessed at *P* < 0.05. Symbols used are as follows: *P < 0.05, ^**^P < 0.01, and ^***^P < 0.001.

## Results

### D-Serine Is Present and Metabolized by the Cochlear Cells

**FIGURE 2:**
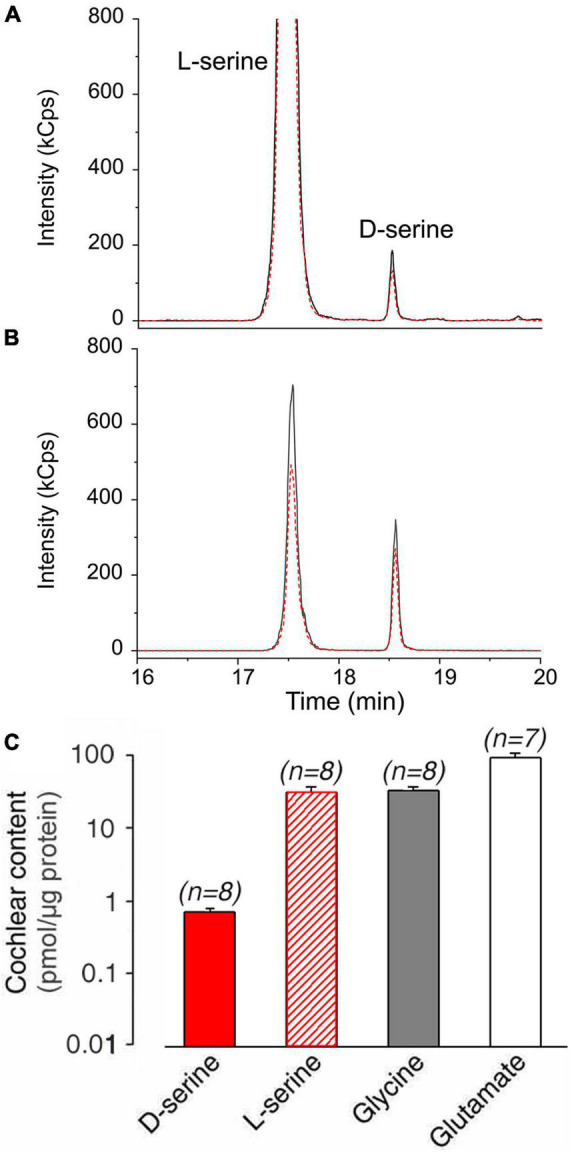
D-serine content in adult mouse cochleae. **(A,B)** Liquid chromatography coupled to tandem mass spectrometry analysis of D-serine content in adult mouse cochlea. Representative LC-MS/MS chromatograms of L- and D-serine in mouse cochlea sample **(A)** and standard **(B)** acquired using LC-MS operating in multiple reaction monitoring (MRM) mode. Quantifier (384.3→162 m/z) and qualifier (384.3→321.7 m/z) transitions are denoted by solid black and dashed red lines, respectively. **(C)** Summary of quantification of D-serine, L-serine, glycine, and L-glutamate expressed in pmol/μg total cochlear protein. All values are expressed as means ± standard error (S.E), (n) number of cochlear specimens.

We first investigated whether D-serine is present in mouse cochlear tissues. Liquid chromatography coupled to tandem mass spectrometry (LC-MS/MS) analysis revealed its presence, along with L-serine, glycine, and L-glutamate. Representative chromatograms of D-serine and L-serine in cochlear samples and the standard are shown in Figures 2A,B. Quantification analyses indicated that the level of D-serine (0.68 ± 0.09 pmol/μg protein; *n* = 8) represented ∼2.3% of L-serine (30.00 ± 5.00 pmol/μg protein; *n* = 8). Levels of cochlear glycine (31.00 ± 4.00 pmol/μg protein; *n* = 8) were ∼45 times higher than D-serine, while L-glutamate (89.00 ± 14 pmol/μg protein; *n* = 7) was by far the most abundant amino acid in the mouse cochlea. These results demonstrated that D-serine is present in the adult mouse cochlea at a relatively low level compared to L-serine, glycine, or glutamate (Figure 2C).

We next examined the cellular distribution of D-serine in the mouse cochleae. We foresaw two possible scenarios. Either D-serine present in some cochlear cells could reflect the site of synthesis, or its presence would correspond to D-serine originating from peripheral sources and taken up by specific cells to be further degraded. In the brain, D-serine is synthetized from L-serine by SR (Wolosker et al., 1999). Intense D-serine immunoreactivity was mainly observed in the cytoplasm of the sensory inner and outer hair cells and their supporting cells (Figure 1A), and of some cells located in the spiral ganglion region (Figure 1B). Serine racemase (SR) immunoreactivity was also pronounced in the cytoplasm of the same types of cochlear cells: i.e., sensory inner and outer hair cells and spiral ganglion neurons, and to a lesser extent, supporting cells of the organ of Corti (Figures 1C,D) of Ser^+/+^ mouse cochleae, but not Ser^–/–^ (Figures 1E,F). These results therefore confirmed the specificity of the polyclonal anti-SR antibody. In rat cochleae, D-serine immunoreactivity was concentrated in IHCs and their supporting cells, and in the supporting cells surrounding the outer hair cells (OHCs) (Figures 1G,H). D-serine was homogeneously distributed in the cytoplasm of most cells, but not in the sensory OHCs (Figures 1H,I). In addition, the immunoreactivities of SR and D-amino acid oxidase (DAAO), the enzyme involved in the degradation of D-serine (Puyal et al., 2006; Pollegioni et al., 2018) were also concentrated in IHCs (Figures 1J,K).

Western-blot analyses revealed the presence of SR in homogenates of rat cochleae and of brain during early development (P0-P10) and an adult stage (P20, Figure 1L). Strikingly, a weak expression of SR was observed during the developmental stages of the cochlea (P0-P5), whereas its expression increased considerably at the onset of hearing (P10; Figure 1L). In addition, we also found SR in adult mouse and guinea-pig cochleae (Figure 1M). We also observed the presence of DAAO in the adult guinea-pig cochlea and brain (Figure 1N). Altogether, these results indicate that D-serine and its synthesis and metabolic enzymes are expressed in the cochlea throughout *Rodentia*, indicating that it might play important functions.

### Genetic Deletion of Serine Racemase Does Not Impair Hearing Function in Mice

**FIGURE 3:**
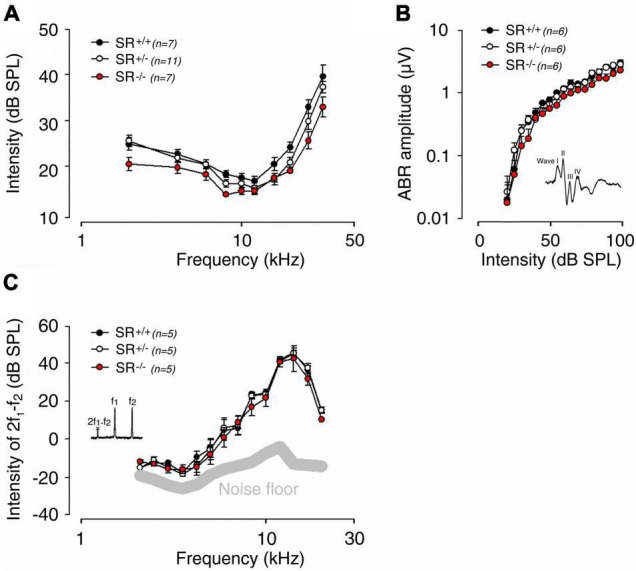
Genetic deletion of serine racemase and hearing in adult mice. **(A)** Auditory brainstem response (ABR) audiograms for SR^–/–^ (*n* = 7), SR^+/–^ (*n* = 11) and SR^+/+^ (*n* = 7) mice obtained by plotting ABR thresholds as a function of the stimulation frequency (2, 4, 6.3, 8, 10, 12.5, 16, 20, 24, and 32 kHz tone bursts). Compare to SR^+/+^ (black circles) mice, SR^–/–^ (red circles) and SR^+/–^ (white circles) mice showed normal sound evoked-response at any stimulating frequency and intensity. **(B)** Normal input-output functions of the ABR wave I evoked by 16 kHz tone-bursts from 0 to 100 dB SPL in SR^–/–^ (*n* = 6), SR^+/+^ (*n* = 6) and SR^+/–^ (*n* = 6) mice. **(C)** Recordings of distortion product otoacoustic emissions (2f_1_-f_2_) reflect the cochlear non-linearity in outer hair cell function. No significant change was measured between SR^–/–^ (*n* = 5), SR^+/+^ (*n* = 5) and SR^+/–^ (*n* = 5) mice. The thick gray line indicates the background noise level of the recording system in the absence of sound. Values are mean ± SE, and n indicates the number of animals tested. There were no statistically significant differences between groups.

To uncover the role of D-serine in hearing function, we next took advantage of SR null mutant (SR^–/–^) mice, which display 85% reduction of D-serine (Basu et al., 2009). Recording of auditory brainstem responses (ABR), which indicated the information transfer into the auditory pathway showed that ABR thresholds and wave I amplitudes in both SR^–/–^ (*n* = 7) and SR^+/–^ (*n* = 11) mice were virtually identical to those of SR^+/+^ (*n* = 7) mice (Figures 3A,B). Distortion-product otoacoustic emissions (DPOAEs), reflecting OHC function, showed no significant difference in amplitude in the three strains (*n* = 5 per strain), suggesting that the function of OHCs is not altered at any of the stimulus frequencies and levels tested (Figure 3C). Taken together, these results indicate that the absence of endogenous D-serine in SR^–/–^ mice does not affect the normal mechanoelectrical transduction of hair cells nor synaptic transmission mechanisms.

### Intracochlear Perfusions of D-Serine Reduce the Amplitude of Cochlear Compound Action Potential

**FIGURE 4:**
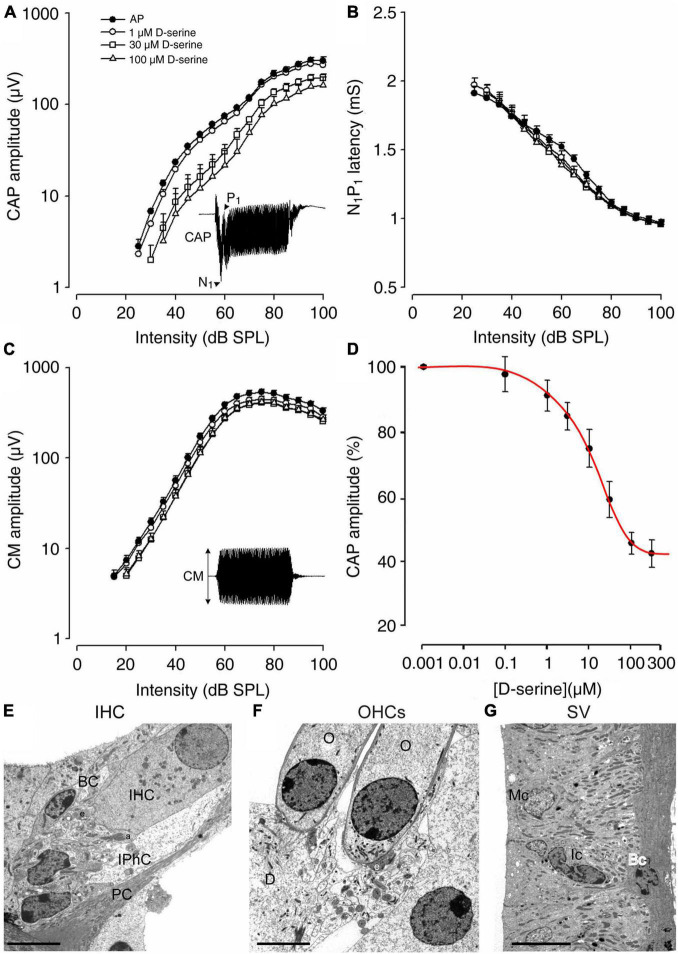
Cochlear functional and morphological consequences of the effects of a 10 min intra-cochlear perfusion of D-serine in adult guinea pig. **(A–C)** Shown are the changes on input–output functions of the compound action potential (CAP), N_1_ latency, and cochlear microphonic (CM) evoked by 8 kHz tone-bursts following either the control perfusion of artificial perilymph (filled circles), 1 μM D-serine (open circles), 30 μM D-serine (open squares), or 100 μM D-serine (open triangles). All points represent mean ± S.E values calculated from five animals. Note that D-serine caused a dose-dependent reduction in CAP amplitude **(A)** but no significant change in N_1_ latency **(B)** or CM **(C)**. **(D)** Shown is a dose-response curve for D-serine. The dose-response data obtained was fitted to a curve using a non-linear least-squares logistic equation. The IC_50_ value for D-serine was calculated to be 75 μM. Note that saturating effects of higher doses of D-serine, i.e., 100 and 300 μM were observed, leading to a limited reduction of CAP. **(E)** Shows a high magnification of the basal pole of the sensory inner hair cell (IHC) surrounding by the border cell (BC) and the inner phalangeal cell (IPhC) close to the pillar cell (PC). Note that the afferent (a) and the efferent nerve endings (e) appear normal. **(F)** Shows a high magnification of normal basal poles of two outer hair cells (O) in contact with one larger efferent terminal (e); Deiters’ cell **(D)**. **(G)** No pathological damage was observed in the organization of the stria vascularis; Marginal cell (Mc); Intermediate cell (Ic); Basal cell (Bc). Scale bars: **(E,F)** = 5 μm, **(G)** = 10 μm.

The pharmacological effects of D-serine on gross cochlear potentials were probed in adult guinea pigs (*n* = 5). The compound action potential of the auditory nerve, which reflects the synchronous activation of the afferent fibers (CAP amplitude: N_1_-P_1_), the N_1_ latency and the cochlear microphonic (CM) that reflects the receptor activity of the OHCs, were recorded after cumulative 10-min intra-cochlear perfusions of increasing doses of D-serine (Figure 4A). We showed that the initial perfusion of artificial perilymph (AP) did not induce significant changes in CAP amplitude or N_1_ latency, nor in CM amplitude (Figures 4A–C). The D-serine effects were therefore compared with the cochlear potentials recorded after this initial control perfusion. D-serine (1–100 μM) induced a dose-dependent reduction in CAP amplitude (Figure 4A), but no significant changes in N_1_ latency or in CM amplitude (Figures 4B,C). To facilitate the comparison of the effects of D-serine concentrations across animals, CAP amplitudes were expressed as the percentage of the control value obtained after the control perfusion at each level (100–40 dB). These percentages were then averaged across levels to provide an overall average percent change for each concentration of D-serine tested. The dose-response data obtained was then fitted to a curve using a non-linear least-squares logistic equation (Figure 4D) revealing an IC_50_ for D-serine of 75 μM. However, a saturation effect of D-serine with higher doses i.e., 100 and 200 μM was observed and produced a maximal reduction in the mean amplitude of the CAP only to 54.4 ± 3.4% and 57.5 ± 4.2% at these two doses, respectively (Figure 4D). Finally, CAP amplitudes returned to control values after washing D-serine out of the cochlea with artificial perilymph (data not shown). Altogether, these results suggest that D-serine may act on the cochlear NMDARs.

Because the suppressing effect of D-serine on CAP could reflect cell damage or excitotoxicity, we next used transmission electron microscopy (TEM) to explore possible ultrastructural changes resulting from intracochlear perfusions of D-serine. As shown in Figures 4E,F, both IHCs and OHCs show a normal morphology and cytoplasm with a well-positioned nucleus, rather apically in the cell body of the IHC (Figure 4E) and basally in the OHC (Figure 4F). In both types of hair cells, mitochondria also had a normal aspect. Both types of hair cells showed well-formed synaptic contacts at their basal poles, the IHCs being contacted by dendrites from type I SGNs (Figure 4E). The OHCs essentially showed one or two large synapses with the axons from the medial efferent system (e in Figure 4F). These efferent synapses showed the classical presynaptic vesicle aggregates and an extended postsynaptic cistern. In addition, no evident ultrastructural abnormality was detected in the stria vascularis (Figure 4G). TEM examination of the cochlea perfused with increasing and cumulative doses of D-serine revealed no structural abnormalities such as damage to sensory hair cells, to the stria vascularis or any early signs of excitotoxicity of auditory-nerve dendrites. These data further support the idea that any functional effect of D-serine on hearing function is physiological, caused by the modulation of fast cochlear synaptic transmission.

### Application of D-Serine Enables Activation of Silent N-Methyl D-Aspartate Receptors in the Cochlea

**FIGURE 5:**
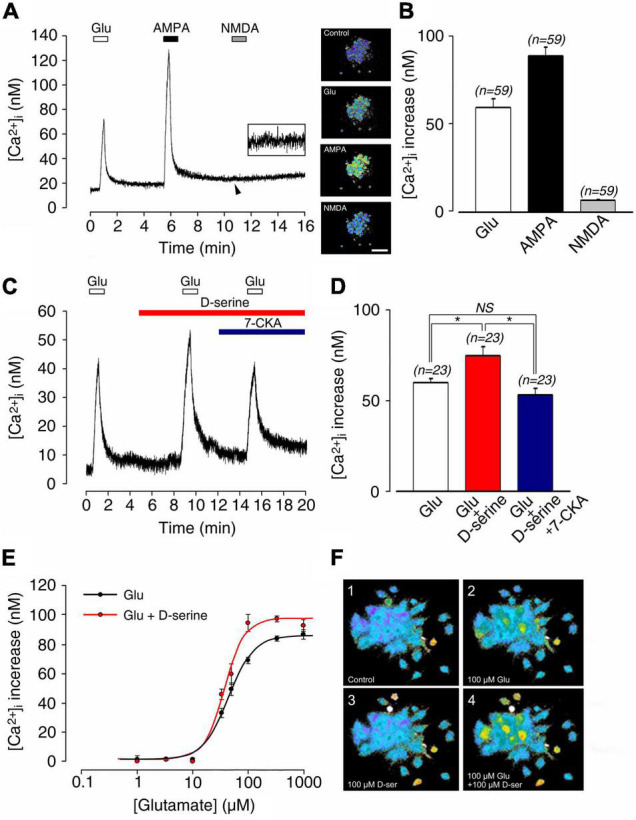
**(A)** Postnatal 1–4 day-old rat spiral ganglion neuron (SGN) somata loaded with Fura-2 AM during sequential pharmacological stimulation with lock buffer containing either 100 μM glutamate, 100 μM AMPA, or 100 μM NMDA. Right inserts: representative images of Fura-2 AM-loaded SGNs before (control condition) and during bath application of glutamate, AMPA, and NMDA. **(B)** Histograms summarizing changes in [Ca^2+^]_*i*_ after stimulation with glutamate (100 μM; *n* = 59), AMPA (100 μM; *n* = 59), and NMDA (100 μM; *n* = 59). **(C)** 100 μM D-serine potentiated glutamate-induced [Ca^2+^]_*i*_ increases (*n* = 23). This effect was blocked by adding the selective antagonist of the glycine site of NMDAR 7-CKA (50μM; *n* = 23) to the bath solution. **(D)** Histograms summarizing changes in [Ca^2+^]_*i*_ after sequential stimulation with 100 μM glutamate alone (*n* = 23), 100 μM glutamate plus 100 μM D-serine (*n* = 23), and 100 μM glutamate plus 100 μM D-serine in presence of 50 μM 7-CKA (*n* = 23). **(E)** Dose-response curve showing the excitatory effects of bath application of 100 μM D-serine on glutamate-induced [Ca^2+^]_*i*_ (glutamate concentration ranging from 1 to 1,000 μM). The calculated EC_50_ for glutamate was reduced from 45.8 μM in the absence, to 35.6 μM in the presence of D-serine (*n* = 212). **(F)** Typical calcium images showing a group of SGNs before (control condition; 1), at the maximum response to 100 μM glutamate (2), at rest in the presence of 100 μM D-serine (3), and finally during the maximum response to 100 μM glutamate plus 100 μM D-serine (4). Inserts **(A)** Scale bar:100 μm. (n) number of neurons, *(*p* < 0.05), (NS) (not significant).

For exploring the modulation of SGN activity, we next used cochlear neural calcium imaging of SGNs loaded with Fura 2-AM (inserts) (Figure 5A). Pharmacological stimulation with glutamate or AMPA, but not NMDA, induced calcium responses in SGNs. Glutamate (100 μM) application in the vicinity of SGN somata transiently increased the resting [Ca^2+^]_*i*_ (59.9 ± 4.9 nM; *n* = 59; Figure 5B). In contrast, a greater increase in [Ca^2+^]_*i*_ of 89.6 ± 4.7 nM (*n* = 59) was observed in the presence of 100 μM AMPA (Figure 5B). Note that NMDA (100 μM) produced only a very marginal increase in [Ca^2+^]_*i*_ of 6.8 ± 0.5 nM (*n* = 59; Figure 5B). These results are consistent with previous reports, demonstrating that fast cochlear synaptic transmission is preferentially mediated by AMPA but not NMDA receptors (Ruel et al., 1999, 2000, 2008; Glowatzki and Fuchs, 2002).

To probe the possible modulatory action of D-serine on cochlear glutamate receptors, SGNs were bathed with classical ionotropic glutamate receptor agonists in the presence or absence of 100 μM D-serine (Figure 5C). Application of D-serine alone in the bathing solution was ineffective in changing [Ca^2+^]_*i*_ (Figure 5C), but significantly increased the glutamate-induced [Ca^2+^]_*i*_ (60.3 ± 2.5 nM vs. 75.6 ± 5.0 nM, respectively; *n* = 23; Figures 5C,D). The effect of D-serine on glutamate responses was blocked by bath application of 7-chlorokynurenic acid (7-CKA, 50 μM), a selective antagonist of the glycine site of NMDAR (Zhu et al., 2016) (53.6 ± 3.5 nM; *n* = 23; Figures 5C,D). Similarly, blockade of D-serine-potentiated glutamate responses was observed in the presence of the competitive glutamate binding site antagonist D-APV (Zhu et al., 2016) (50 μM; *n* = 25; not shown).

**FIGURE 6:**
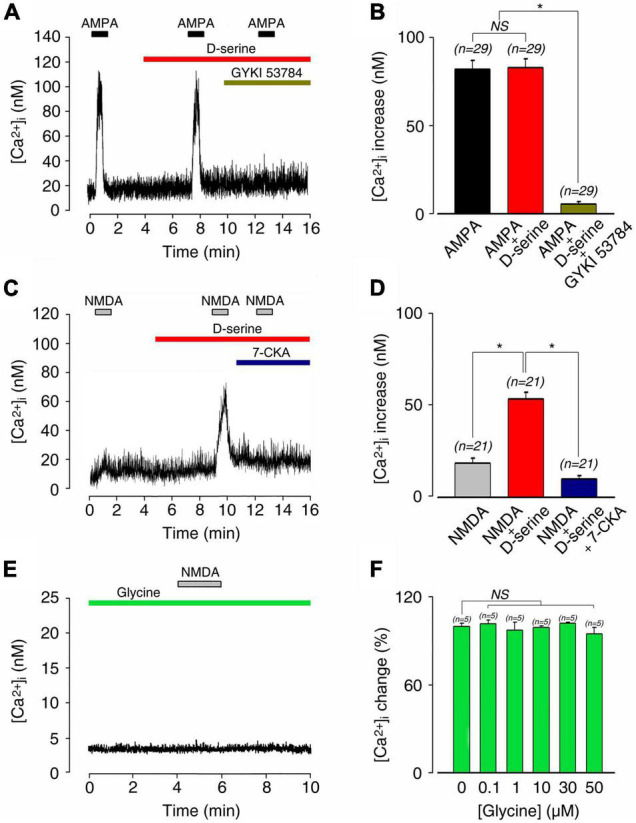
D-serine and not glycine enabled NMDA-induced [Ca^2+^]_*i*_ responses on postnatal 1–4-day rat SGNs. **(A)** AMPA-induced [Ca^2+^]_*i*_ increases (100 μM; *n* = 29) were unaffected by the presence of 100 μM D-serine (*n* = 29). The pharmacological specificity of the AMPAR stimulation was verified by using the selective stereoisomer AMPAR antagonist GYKI 53784 (40 μM; *n* = 29). **(B)** Histograms summarizing changes in [Ca^2+^]_*i*_ after a sequential stimulation with 100 μM AMPA alone (*n* = 29), 100 μM AMPA plus 100 μM D-serine (*n* = 29), and in the presence of GYKI 53784 (*n* = 29). **(C)** Co-application of 100 μM D-serine with 100 μM NMDA unmasked a large [Ca^2+^]_*i*_ increase, leading to an increase of 540% compared to NMDA alone (*n* = 21). Pharmacological blockade of the D-serine excitatory effect was suppressed by using the selective NMDAR antagonist 7-CKA (50 μM; *n* = 21). **(D)** Histograms summarizing changes in [Ca^2+^]_*i*_ after a sequential stimulation with 100 μM NMDA alone (*n* = 21), 100 μM NMDA plus 100 μM D-serine (*n* = 23), and in the presence of 7-CKA (50 μM; *n* = 23). *(*p* < 0.05). **(E)** 100 μM NMDA in the presence of 20 μM glycine did not elicit any response or basal [Ca^2+^]_*i*_ changes (*n* = 28). **(F)** Increasing concentrations of glycine (0.1, 1, 10, 30, 50 μM) did not change basal [Ca^2+^]_*i*_ (*n* = 25) in SGN somata compare to the control condition. (n) number of neurons, *(*p* < 0.05), (NS) (not significant).

As shown in Figures 5E,F, dose-response curves of glutamate-induced calcium responses obtained in the presence or absence of 100 μM D-serine revealed that the amino acid significantly increased the responses compared to glutamate alone. Thereby, EC_50_ for glutamate was reduced from 45.8 μM in the absence, to 35.6 μM in the presence of D-serine (*n* = 212; Figure 5E). By contrast, the AMPA (100 μM)-induced increase of [Ca^2+^]_*I*_ (81.3 ± 5.0 nM; *n* = 29) was not affected by the presence of 100 μM D-serine (82.3 ± 4.6 nM; *n* = 29; Figures 6A,B). The pharmacological specificity of the AMPAR stimulation in the presence of D-serine was further verified by blocking the AMPA-induced response with the selective AMPAR antagonist GYKI 53784 (Ruel et al., 2000) (40μM; Figures 6A,B). These data suggest that the potentiating effect of D-serine on glutamate responses is most likely mediated by turning on silent NMDAR.

To further examine this scenario, we next investigated the effect of D-serine on NMDA-evoked calcium responses. When applied alone, both NMDA and D-serine did not evoke a significant [Ca^2+^]_*i*_ response (Figures 6C,D). In contrast, co-application of D-serine (100 μM) with NMDA (100 μM) evoked a strong increase in [Ca^2+^]_*i*_ (57.7 ± 3.6 nM; *n* = 21) compared to NMDA alone (14.7 ± 1.5 nM; *n* = 21; Figures 6C,D). The pharmacological specificity of the D-serine to modulate NMDAR was confirmed by blocking its potentiating effects with 7-CKA (50 μM; *n* = 21; Figures 6C,D). Finally, because NMDAR could be also sensitive to glycine, which can act as a putative co-agonist, we tested the effect of glycine. Application of NMDA (100 μM) in the presence of 20 μM glycine never induced calcium responses in SGNs in the 28 neurons tested. Moreover, increasing concentrations of glycine (0.1–50 μM) did not change basal [Ca^2+^]_*i*_ (Figures 6E,F). We thus concluded that cochlear D-serine, but not glycine, unsilences NMDAR responses located on SGN somata.

### Mice With an Serine Racemase Deletion Are Protected From Noise-Induced Permanent Hearing Loss

**FIGURE 7:**
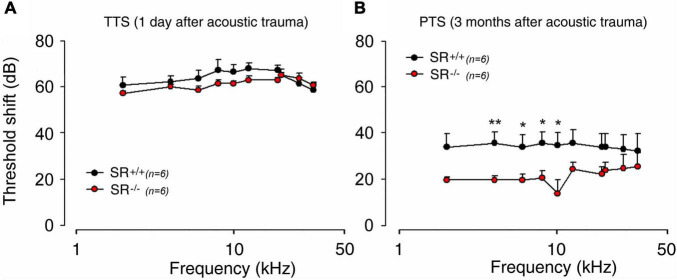
Serine racemase deletion improved hearing recovery after noise exposure in adult mice. **(A,B)** ABR threshold shift in the SR^+/+^ (black filled circle) and SR^–/–^ (red filled circle) measured 1 day **(A)** and 3 months **(B)** after exposure to an 4–8 kHz octave-band noise at 100 dB for 2 h. Note that the threshold shift was similar 1 day after noise exposure in both strains. Interestingly, a better recovery of hearing ∼16.5 dB was observed in SR^–/–^ mice 3 months after noise exposure. *(*p* < 0.05) and **P < 0.01.

Because aberrant NMDAR activation has been consistently found to contribute to trauma-induced cochlear damage (Guitton et al., 2003; Ruel et al., 2008; Bing et al., 2015; Sanchez et al., 2015), we next examined the role of D-serine in noise-induced hearing loss (NIHL). Acoustic overstimulation sharply increases ABR thresholds (temporary threshold shift, TTS) equally in both SR^–/–^ (mean TTS: 64.8 ± 1.2 dB) and control SR^+/+^ (mean TTS: 60.4 ± 0.7 dB) mice; regardless of the tested frequency (Figure 7A). In contrast, threshold recovery at the frequencies from 4 kHz to 10 kHz was significantly better (*p* < 0.05 or 0.01) in SR^–/–^ (mean PTS: 18.7 ± 1.5 dB) than SR^+/+^ (mean PTS: 35.2 ± 0.4 dB) mice (Figure 7B). These results indicate that SR^–/–^ mice displayed greater capacity for recovery from NIHL than WT, suggesting that deletion of SR might favor the functional recovering of the cochlear nerve endings of SGNs from acoustic damage by preventing the harmful accumulation of D-serine and subsequent over-activation of NMDARs.

## Discussion

How NMDARs in the adult cochlea are activated remains enigmatic. In the present study, we report for the first time that D-serine, a well-known endogenous co-agonist of NMDAR, is metabolized by SR and DAAO in the cochlear sensory IHCs and their supporting cells, in the SGNs and glial cells of several rodent species. In addition, we reveal that this amino acid is a necessary for the full NMDAR activation at the IHC-SGNs complex. Importantly, we demonstrate that D-serine plays an important role in promoting long-term cochlear damage following acoustic trauma.

Although the role of D-serine at synapses in the CNS has been studied in detail, except for the retina its function in sensory organs and in information processing remains remarkably poorly explored. Using LC-MS/MS, we established that the steady background level of D-serine in the adult cochlea is relatively low (0.680 nmol/mg protein) compared to L-glutamate, L-serine, or glycine. This is ∼10 times lower than the average retrieved levels in the adult mouse brain, but still comparable to the levels found in the retina (Stevens et al., 2003), where D-serine has been found to fully activate NMDARs. Although present at relatively low level in the adult mammalian cochlea, our bulk measurements do not reflect local amounts in the vicinity of the synaptic cleft and thus preclude any conclusion about the functional ability of D-serine to putatively activate NMDAR under physiological conditions. Nevertheless, the involvement of D-serine could be critical in pathological conditions such as excitotoxicity and acoustic trauma-induced upregulation of GluN1 mRNAs and overexpression of the heavy C2’ form of GluN1 and GluN2B subunits, respectively (Puel, 1995; Eybalin et al., 2010). Thus, such a local amount of D-serine could induce long-term deleterious effects on primary auditory neurons.

Our immunolabeling revealed that D-serine populates the cytoplasm of different cells populations inside the organ of Corti, including the sensory IHCs, pillar cells, Deiters’ cells, and the cytoplasm of some SGNs and their glial cells that also express SR. These observations indicate that D-serine is a metabolite produced by the cochlea tissue itself, but also demonstrate that D-serine is produced at some specific sites where it could reach threshold concentration to activate NMDARs. SR immunoreactivity was also pronounced in the spiral limbus, in the myelin enveloping the auditory neurons, and in their glial cells, suggesting that SR in glia may play specific roles related to inflammatory insults (Perez et al., 2017; Ivanov and Mothet, 2019). Therefore, D-serine and SR are localized in the same subsets of cochlear cells, except the SGNs, where SR is absent. This suggests that these latter cells may uptake (and package) the amino acid, but do not have the ability to synthesize it. These results are consistent with a previous study that showed expression of D-serine and SR in rat vestibular sensory epithelia (Dememes et al., 2006), but also support the idea of a metabolic compartmentalization in which some cells have the ability to synthesize and release D-serine, while others reuptake it and degrade it through the intervention of DAAO. Pillar and Deiters’ cells are supporting cells that provide a structural scaffold to enable mechanical stimulation of sensory hair cells, but also actively regulate the homeostasis of ion composition of the perilymph and endolymph (Wan et al., 2013). Therefore, the presence of D-serine in these supporting cells suggests that D-serine may be secreted into and taken up from inner-ear fluids. D-Serine is transported by five amino acid transporters, SLC1A4/ASCT1, SLC1A5/ASCT2, SLC7A10/Asc-1, Slc38a1/2/SNAT1/2 in the brain (Rosenberg et al., 2013; Foster et al., 2016) and the SLC6A14/ATB0^+^ in the intestine (Ruffin et al., 2020). Intriguingly, many of these transporters are expressed by cochlear cells (Espino Guarch et al., 2018). However, whether these transporters are involved in regulating the levels of D-serine in the cochlea is not known and awaits future studies.

Strikingly, we observed that the expression of SR coincides with the development of the cochlea, with levels being low (or negligible) at P0–P5, while reaching its peak at the onset of hearing (∼P10). These observations suggest that D-serine might play a major role in the development and maturation of the hearing system during postnatal development and adult stages. Nevertheless, analysis of SR^–/–^ mice showed no obvious sensory deficit, which could reflect the persistent residual concentration of D-serine that has been previously described in SR^–/–^ mice (Miyoshi et al., 2012).

We demonstrated that D-serine and SR were mainly localized at the level of the IHC-synaptic complex. Together, these results suggest that D-serine may play a functional role in the modulation of cochlear NMDARs. In the cochlea, NMDARs play an important neurotrophic function in neuronal regeneration and synapse formation in response to excitotoxic injury (D’Aldin et al., 1997), and a critical role before hearing onset (Zhang-Hooks et al., 2016). Previous studies showed that the NMDAR subunits GluN1 and GluN2A-D (Safieddine and Eybalin, 1992; Kuriyama et al., 1993; Niedzielski and Wenthold, 1995; Usami et al., 1995), but not GluN2A, are expressed in mature cochleae and localized at the postsynaptic density of sensory IHC synapses (Ruel et al., 2008). Also, several other studies have shown that application of NMDA in presence of glycine in Mg^2+^ free solution failed to elicit detectable current on SGN somata or a change in calcium concentration in postsynaptic afferent boutons (Nakagawa et al., 1991; Ruel et al., 1999, 2008; Glowatzki and Fuchs, 2002).

Multiple intracochlear perfusions of D-serine in adult guinea pigs *in vivo* allowed us to study the functional impact of an increase in the amount of D-serine at the level of the IHC-synaptic complex. A consistent decrease in CAP amplitude in response to sound during intracochlear perfusions of cumulative and increasing doses of D-serine, without affecting the receptor potentials, indicated that D-serine acts on the auditory nerve terminals, leaving sensory hair-cells and the stria vascularis unaffected. The CAP is an indirect monitor of auditory-nerve activity, as a decrease in its amplitude indicates that fewer auditory nerve fibers discharge in synchrony in response to a tone. This situation could be due to either inhibition or excitation in the spontaneous (asynchronous) firing of single auditory neurons (Ruel et al., 2001).

Finally, a decrease or even an abolition of the CAP amplitude may be due to the development of cochlear excitotoxicity observed during perilymphatic perfusions of cumulative doses of glutamate receptor agonists (Puel et al., 1991b). Close electron microscopic examination of cochleae perfused *in vivo* with increasing and cumulative doses of D-serine did not reveal any cochlear structural damage or any early signs of excitotoxicity of the auditory nerve endings below the sensory inner hair cells. These results are not in agreement with our previous neurotoxicity experiments made from *CA1* pyramidal cells in the hippocampal *in vitro* slice preparation, demonstrating that activation of D-serinergic NMDARs is responsible for acute excitotoxicity and neuronal cell death (Papouin et al., 2012). This major difference could be explained by a different density and distribution of NMDAR at the cellular level.

To determine the mechanism, i.e., decrease or increase of auditory neuronal firing, by which D-serine modulates auditory gross cochlear responses, calcium imaging experiments on neonatal rat spiral ganglion neurons were performed. In the prehearing cochlea, spiral ganglion neurons contain functional NMDA receptors that prolong synaptic currents, enhance postsynaptic depolarization and the probability of action potential generation (Zhang-Hooks et al., 2016). A developmental switch of NMDAR subunits from GluN2B to GluN2A is a common feature among CNS neurons (Cull-Candy et al., 2001; Futai et al., 2001; Liu et al., 2004), but this feature is not always shared in the lower auditory pathway (Puel, 1995; Ruel et al., 2008). During cochlear development, changes in subunit composition and density of NMDARs could alter the affinity for D-serine, as demonstrated in hippocampal slices (Le Bail et al., 2015). Finally, NMDAR-mediated currents can be induced by chemical stimuli such as salicylate in neonatal spiral ganglion neurons (Peng et al., 2003; Ruel et al., 2008), suggesting the presence of a latent pool of NMDAR in the immature and mature cochlea.

Focal application of D-serine in the vicinity of the somata of spiral ganglion neurons greatly potentiated the glutamate response. The D-serine-enabled glutamate response could be mimicked by the application of NMDA in the presence of D-serine and was selectively blocked by 7-chlorokynurenic acid (7-CKA), a competitive antagonist at the glycine binding site of NMDARs (Kemp et al., 1988). Similar results were obtained in the presence of the competitive NMDAR antagonist D-AP5, which binds at the glutamate site of the GluN2 subunit.

We draw three conclusions from these functional data. First, they show that the glycine binding site is not saturated. Second, they show that D-serine, but not glycine, enables cochlear NMDA excitatory responses by acting specifically at the co-agonist site of the GluN1 subunit and that of the NMDARs. Third, the NMDARs on which D-serine is acting are canonical GluN1-GluN2 NMDARs. Therefore, the decrease in CAP amplitude observed during perilymphatic perfusions of D-serine could be explained by an excitatory effect of D-serine on single auditory nerve fibers unable to respond in synchrony to the sound stimulation. In contrast, glycine was ineffective in unmasking NMDAR responses, and did not modify the level of basal [Ca^2+^]_*i*_ up to concentrations of 50 μM. These results are in agreement with those of Puel et al. (1991a) showing that intracochlear perfusions of Mg^2+^-free perilymph containing 100 or 1000 μM glycine have no effect on gross cochlear potentials. Together, our results suggest that D-serine unmasks silent “D-serinergic” NMDARs in the mammalian cochlea. Here, we demonstrated that SR suppression in mice improves hearing recovery from noise-induced PTS compared to WT mice. These results suggested that D-serine is involved in noise-induced cochlear injuries, probably through potentiating cochlear NMDARs. Indeed, glutamate and NMDARs may have an important neurotrophic function in neuronal regeneration and synapse formation in response to excitotoxic injury, comparable to noise-induced trauma injury in the guinea-pig cochlea (D’Aldin et al., 1997). During this re-innervation process, a clear overexpression of the GluN1 subunit was observed in SGNs (Puel, 1995), which could reflect changes in the density of NMDAR or of subunits constituting channel complexes, increasing neuronal excitability. Molecular studies also suggest changes in the electrophysiological properties of NMDARs, such as an increase in calcium permeability during neuronal plasticity in adult brain (Pellegrini-Giampietro et al., 1991). This greater excitability of SGNs enables the cochlea to promote neurite regeneration and synapse formation after noise trauma. On the other hand, changes in the density and functional properties of cochlear NMDARs could create neuronal vulnerability to injury in the critical periods observed during neural plasticity and development (Ikonomidou et al., 1989; Mcdonald et al., 1990). Further studies are now necessary to distinguish the protective mechanisms against noise trauma observed in SR^–/–^ mice.

## Conclusion

Aberrant NMDAR activation and related auditory nerve excitation are suspected factors of cochlear tinnitus induced by salicylate or noise exposures (Guitton et al., 2004; Van De Heyning et al., 2014; Bing et al., 2015). Accordingly, NMDAR inhibition is a promising pharmacological approach for the treatment of synaptopathic tinnitus (Bing et al., 2015). Here, we discover that D-serine is a necessary component of full NMDAR activation in the cochlea. These results may have important clinical implications by offering new opportunities in the modulation of NMDARs, to prevent some cochlear pathologies such as auditory neuropathy.

## Data Availability Statement

The raw data supporting the conclusions of this article will be made available by the authors, without undue reservation.

## Ethics Statement

The animal study was reviewed and approved by the European and French Directives on Animal Experimentation and with Local Ethical Committee Approval (agreements C75-05-18 and 01476.02, license #6711). Written informed consent was obtained from the owners for the participation of their animals in this study.

## Author Contributions

JR supervised the project. JW and FF performed immunocytochemistry, Western blot, and noise-damage experiments. JR and NS performed *in vivo* electrophysiological experiments. NS contributed to TEM and Western blot experiments. CL and JS together with J-PM performed the dosage of amino acids by LC-MS/MS. JR, JW, and J-PM wrote the manuscript. JR, JW, J-PM, JS, and J-LP reviewed the manuscript. All authors read and approved the final manuscript.

## Conflict of Interest

The authors declare that the research was conducted in the absence of any commercial or financial relationships that could be construed as a potential conflict of interest.

## Publisher’s Note

All claims expressed in this article are solely those of the authors and do not necessarily represent those of their affiliated organizations, or those of the publisher, the editors and the reviewers. Any product that may be evaluated in this article, or claim that may be made by its manufacturer, is not guaranteed or endorsed by the publisher.
