# Supplementary material for: Physiopathological Relevance of D-Serine in the Mammalian Cochlea
Source: Front Cell Neurosci. 2021 Dec 17;15:733004. doi: 10.3389/fncel.2021.733004 (PMC8718999; doi:10.3389/fncel.2021.733004)
Supplement: Supplementary file 1 [file Data_Sheet_1.docx]

**Additional file 1**

**Supplemental Data**

**Figure S1**


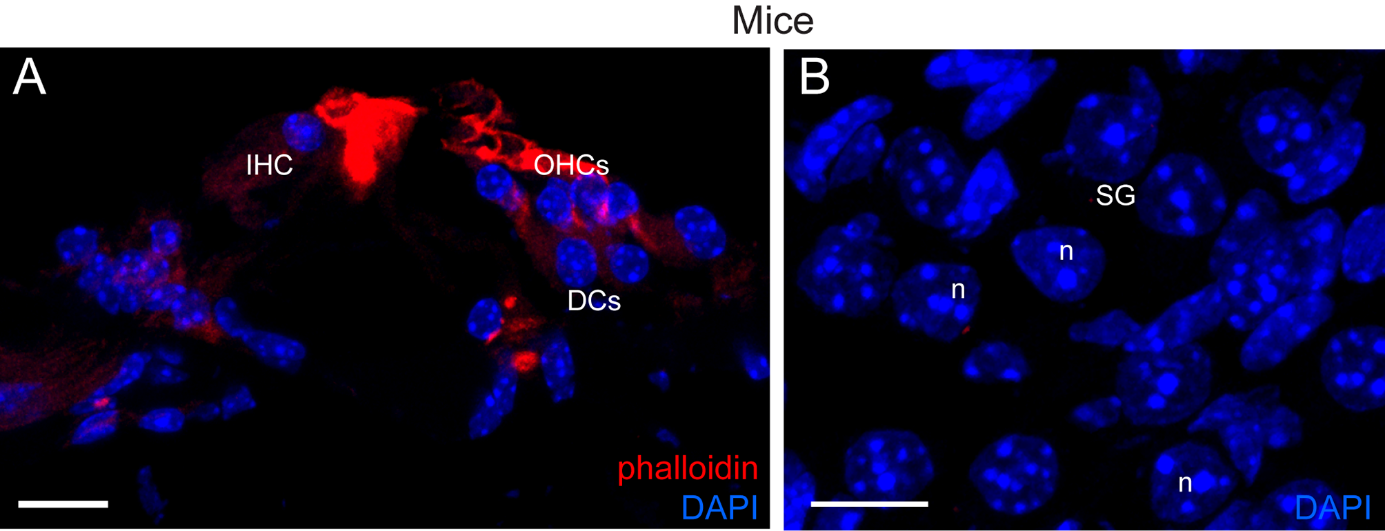


**Figure S1. Control staining**

**A**-**B**: confocal images of cryostat sections of the organ of Corti (**A**), spiral ganglion (**B**) from the adult mice. The sections were incubated with FITC-conjugated donkey anti-goat IgGs or FITC-conjugated donkey anti-rabbit IgGs and stained with Phalloidin rhodamin to labelled actin (red) and counter-stained with Hoechst (blue). Note no FITC fluorescence signals was observed. DCs: Deiter’s cells, IHC: inner hair cell, OHCs : outer hair cells. SG: spiral ganglion, n: neuron. Scale bars: A=10 µm, B=20 µm.

**Figure S2**


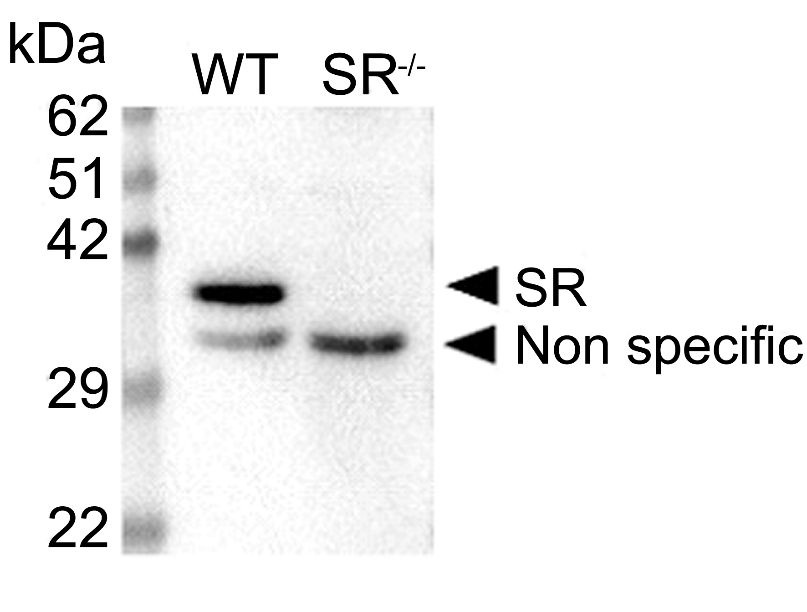


**Figure S2.** **Demonstration of the specificity of the polyclonal anti-SR antibody using SR-null mutant mice.** Protein brain extracts (40 µg/lane) from SR^-/-^ and WT control littermates mice (2 months old) were subjected to electrophoresis (10% SDS-polyacrylamide gel) and electroblotted onto PVDF membrane. Immunoblot reveals the presence of a band for SR at the expected 37 kDa in the WT but not in the SR^-/-^ mice.
